# Treat and release: an observational study of non-conveyed high-acuity dispatches in a Danish emergency medical system

**DOI:** 10.1007/s11739-024-03618-3

**Published:** 2024-05-15

**Authors:** Signe Amalie Wolthers, Tor Jerker Mikaelsson, Mathias Geldermann Holgersen, Stig Nikolaj Fasmer Blomberg, Lars Bredevang Andersen, Søren Mikkelsen, Helle Collatz Christensen

**Affiliations:** 1 Emergency Medical Services, Prehospital Center, Region Zealand, Ringstedgade 61, 13 Floor, 4700 Næstved, Denmark; 2 Department of Clinical Medicine, University of Copenhagen, Copenhagen, Denmark; 3 Department of Paediatrics and Adolescent Medicine, Paediatric Pulmonary Service, Copenhagen University Hospital, Rigshospitalet, Blegdamsvej 9, 2100 Copenhagen, Denmark; 4 Prehospital Research Unit, Department of Anaesthesiology and Intensive Care, Odense University Hospital, Kildemosevej 15, 5000 Odense, Denmark

**Keywords:** Epidemiology, Non-conveyance, Treat and release, Patient safety, Emergency medical services

## Abstract

Non-conveyance refers to the practice of treating a patient on-site without transporting them to a medical facility. It may decrease unnecessary hospital transfers and improve patient satisfaction. Nonetheless, ensuring patient safety remains paramount. The objective of the study was to assess admission to hospital and mortality in non-conveyed patients. This population-based cohort study included all high-acuity dispatches in Region Zealand, Denmark between 2019 and 2022. The primary outcome was admission within 48 h, and the secondary outcome was 30-day mortality. Descriptive statistical analyses were conducted, and logistic regression models were used to estimate adjusted odds ratios and 95% confidence intervals. A non-conveyance rate of 14% was identified in 95,238 transports. Admission within 48 h was seen in 22% of non-conveyed patients vs. 95% in conveyed patients, *p* < 0.001. The adjusted analysis showed a decreased likelihood of admission within 48 h within non-conveyed patients, with an aOR of 0.01 95% CI (0.01–0.01). Non-conveyed patients had a crude 30-day mortality rate of 2 vs. 6% among conveyed patients, *p* < 0.001. The adjusted analysis showed an increased likelihood of 30-day mortality in non-conveyed patients with an odds ratio of 1.21, 95% CI (1.05–1.40). Non-conveyed patients constitute a substantial proportion of patients assessed by ambulances following high-acuity dispatch. Less than one in four non-conveyed patients were admitted within 48 h. Despite the low crude mortality in this study, an increased likelihood of mortality was found within the non-conveyed population. However, additional investigation is warranted in future research.

**Supplementary Information:**

The online version contains supplementary material available at 10.1007/s11739-024-03618-3.

## Introduction

Throughout the latest decades, global healthcare systems have reported an escalating number of emergency department (ED) contacts, leading to significant overcrowding and potential compromises in patient safety [1, 2]. In response to this growing demand, a shift towards more adaptive Emergency Medical Services (EMS) systems has arisen. In this context, non-conveyance, a practice where patients receive on-site care or assessment without hospital transfer, has received a growing emphasis as a potential solution to alleviate some of these challenges [2–7]. Such practice reflects a broader transition of the EMS systems of industrialised countries, evolving from focusing on patient transport to delivering advanced prehospital care [2]. Yet these findings may not apply to all settings and regions, as there may be regional differences.

In Denmark, legal mandates have specified guidelines for ambulance personnel when deciding to release patients at the scene [8]. These non-conveyed patients undergo on-scene treatment, receive discharge without subsequent follow-ups, or are redirected to primary healthcare facilities. Yet, non-conveyance has implications for the efficiency and availability of EMS systems, and concerns regarding patient safety have been raised. In a scoping review on patient safety, the predominant causes of litigations were lack of assistance (26%) followed by delays or failures in treatment (18%), hospital admissions (12%) and diagnoses (11%), all of which could be related to non-conveyance decisions [9]. However, data within this field are limited [10], and previous research on non-conveyance has reported contradictory results [2, 6, 11].

Such disparities may arise from differing surrogate measures for patient safety: whereas some studies highlight ED contacts, others prioritize mortality or on-site patient vitals [4]. In addition, inconsistent research designs, definitions of non-conveyed populations, and regional variations among EMS systems complicate the field further. Finally, legislative nuances play a role; certain EMS systems have established guidelines for non-conveyance [3], whereas others remain ambiguous [12, 13]. Collectively, these inconsistencies challenge the direct comparison of available evidence. This is in part reflected in the large variations among reported non-conveyance rates, ranging from as low as 3.7% to apparent 93.7% [2].

When an ambulance is dispatched with lights and sirens for a life-threatening situation, it is surprising how the transport can end as non-conveyed, and this causes reflection on the nature and occurrence of these missions. Given these considerations and the critical patient safety implications of non-conveyance decisions, an improved understanding is essential. Hence, this study aimed to investigate hospital admissions and mortality outcomes associated with non-conveyance in high-acuity dispatches within Region Zealand, Denmark.

## Methods

### Study design

This study was a population-based retrospective cohort study with a 30-day follow-up. Data were derived from the emergency medical dispatch centre (EMDC) on severe and immediate high-acuity calls of category A (lights and sirens) between January 1, 2019, and September 30, 2020, in Region Zealand, Denmark.

### Setting and population

Region Zealand covers an area of 7274 km^2^ and has approximately 840,000 inhabitants. The population density is 116 inhabitants/km^2^. The Danish healthcare model is funded by direct and indirect taxes. The Danish EMS is organized in a three-tiered system: an ambulance staffed with two paramedics, a rapid response vehicle with a paramedic and a physician-staffed ground-based or helicopter-based mobile emergency care unit [14, 15]. The EMDC is staffed by medical professionals, predominantly nurses and paramedics. Medical dispatchers employ a criterion-based decision tool to dispatch prehospital resources and determine the urgency of the response [16]. The decision tool is divided into chief complaint categories; each categorized into more specific complaint groups and assigned a unique code. There are four levels of emergency that will require ambulance dispatch: A: Life-threatening or potentially life-threatening condition, immediate response required (high-acuity dispatch with lights and sirens), B: Urgent, but not life-threatening condition, C: Non-urgent condition that needs ambulance care, D: Non-urgent supine patient transport. When paramedics encounter a patient suitable for non-conveyance, the medical control physician is always contacted to confer and consolidate the decision to let the patient stay at home. If the physician at the control centre disagrees, the patient must be transported to a hospital for evaluation.

All patients assessed following a high-acuity dispatch were eligible for inclusion encompassing all age groups. Patients without valid personal identification numbers were excluded, as this was necessary for coupling with other registries [17]. All Danish citizens are assigned a unique personal identification number [17, 18]. Software from EMDC provided data on high-acuity calls within the study period.

### Measures

Data on the personal identification number, day of week and time of day of the incident, ambulance response time, on-scene time and Danish Index criteria code were collected. Calls where the prehospital care provider chose not to convey a patient were manually identified. Additionally, non-conveyed calls were cross-referenced with the Danish National Patient Registry using the patients’ unique personal identification number [17, 18] to ascertain any subsequent hospital contact. Discharge diagnoses registered at the hospital contact and admissions to the hospital were coupled to the cases. Additionally, patients admitted within 3 h, regardless of conveyance, were identified, along with patients discharged within six hours from admission were identified. Finally, mortality was recorded from the Civil Registration System Registry [18]. Patients were considered admitted once they had a contact in the hospital, whether this was an ED contact where the patient was under evaluation or admitted to a specialised unit for a longer period of time.

### Outcomes

The primary outcome was admission to the hospital within 48 h following dispatch. The secondary outcome was 30-day mortality. Further, admission within three hours and demographic characteristics among the included patients were assessed.

### Analyses

Descriptive statistics were reported as absolute numbers and percentages or medians and interquartile ranges. Comparative analyses were performed using non-parametric testing to examine subgroups as appropriate. Categorical data were analyzed using Fisher’s exact test. Multivariable regression models were used to estimate adjusted odds ratios (aOR) and corresponding 95% confidence intervals (CI). The analyses were adjusted for prognostic factors, including mortality, admission within 48 h, response time, age, sex, and chief complaints. A directed acyclic graph was provided to illustrate the basis of the included variables [19]. Patients who were terminally ill or sustained cardiac arrest were included in all analyses. Age was stratified according to 30-year intervals. The chief complaint was stratified into categories with > 1000 patients. Response time was defined as the time from the initial call to the dispatch centre to the arrival of the first EMS personnel. There was no imputation of missing data. Statistical significance was considered at a two-sided *p*-value of <0.05; all statistical tests were performed using R version 4.1.3 (2022-03-10).

## Results

**Fig. 1:**
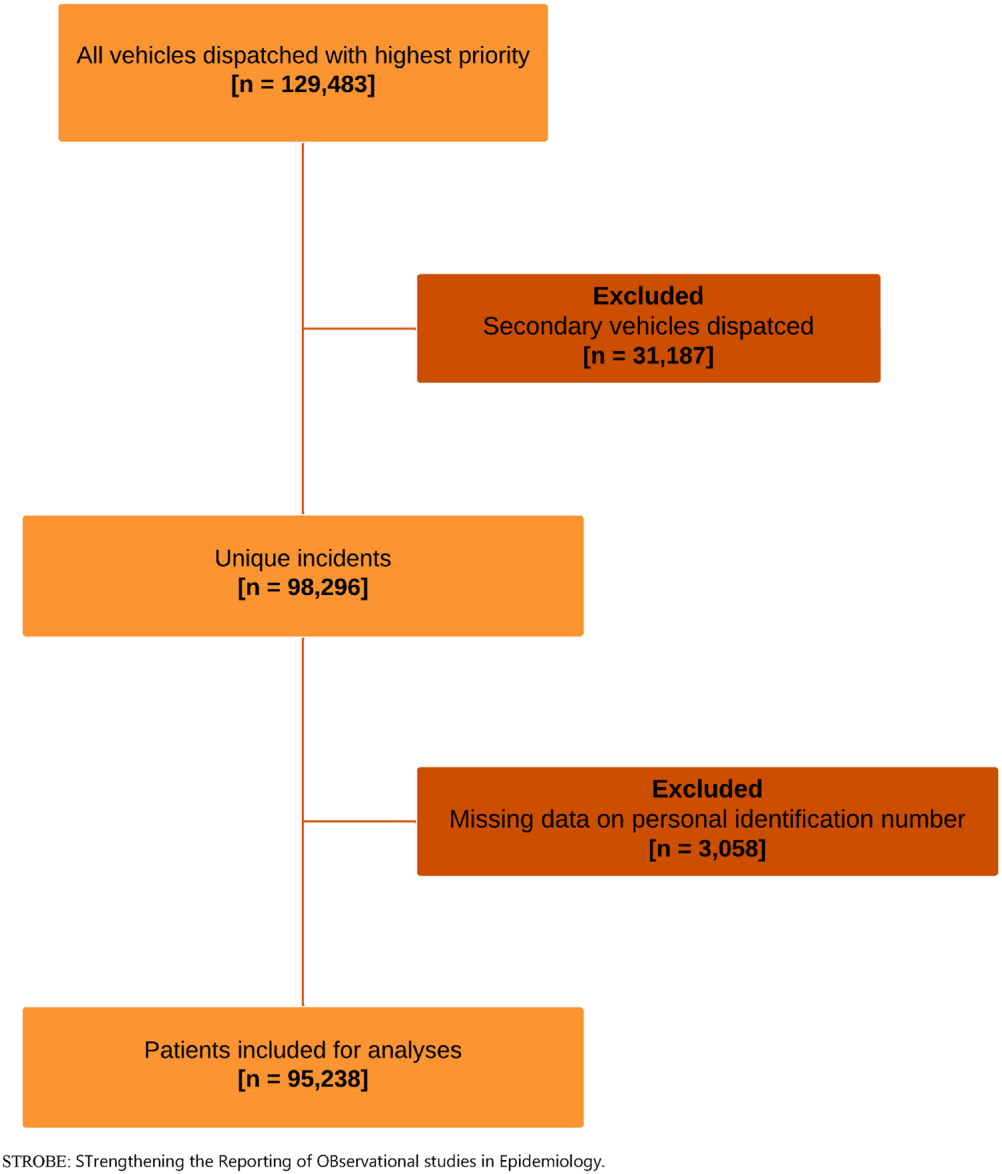
Revised STROBE flow chart of inclusion

A total of 129,483 ambulances were dispatched as category A and 31,187 (24%) of these dispatches were excluded as being dispatched as secondary units. This excluded no patients or conveyances. Due to the lack of a personal identification number, 3058 incidents were excluded from the study. This left 95,238 incidents for inclusion, of whom 69,273 were unique patients (Fig. 1). A total of 13,451 of all high-acuity responses resulted in non-conveyance, corresponding to a non-conveyance rate of 14% (95% CI 13.90–14.36).

### Baseline demographics

Non-conveyed patients were significantly younger than conveyed patients, with a median age of 55 years [interquartile range (IQR) (31–74)] compared to 65 years [IQR (44–77)] (*p* < 0.001). Furthermore, non-conveyed patients experienced a shorter response time until the ambulance arrived, with a median time of 7:04 min [IQR (4–11)] compared to 7:48 min [IQR (4–11)]. Males accounted for 55% of both groups, *n* = 7333 in the non-conveyed group compared to *n* = 45,157 in the conveyed group. There was no significant difference between males and females regarding conveyance decisions, *p* = 0.17. The on-scene time was significantly longer for non-conveyed patients, with a median time of 35:00 min [IQR (25–48)] compared to 22:09 [IQR (16–22)] for conveyed patients, *p* < 0.001. The most common chief complaint was “chest pain”, which accounted for 2972 cases (22%) of non-conveyed patients compared to 19,147 cases (23%) of conveyed patients *p* < 0.001. In 1549 cases (12%) of non-conveyed patients, “impaired consciousness” was the dispatch criterion as compared to 13,129 (16%) of the conveyed patients, (*p* < 0.001). “Unclear problems” accounted for 2565 cases (19%) among non-conveyed patients compared to 11,048 cases (14%) among conveyed patients, *p* < 0.001 (Table 1).

**Table 1:** Baseline characteristics of conveyed and non-conveyed patients

	Conveyed patients, *N* = 81,787^a^	Non-conveyed patients, *N* = 13,451^a^	*p*-value^b^	Overall, *N* = 95,238^a^
Age (years)	65 (44, 77)	55 (31, 74)	<0.001	63 (42, 77)
Missing	88	32		120
Sex			0.17	
Female	36,550 (45%)	6091 (45%)		42,641 (45%)
Male	45,157 (55%)	7333 (55%)		52,490 (55%)
Missing	80	27		107
Response time (minutes)	7 (4, 11)	7 (4, 11)	<0.001	7 (4, 11)
Missing	173	0		173
Time on scene (minutes)	22 (16, 30)	35 (25, 48)	<0.001	26 (16, 30)
Time of the day				
Day	42,554 (52%)	6207 (46%)	<0.001	48,761 (51%)
Evening	25,874 (32%)	4657 (35%)	<0.001	30,531 (32%)
Night	13,359 (16%)	2587 (19%)	<0.001	15,946 (17%)
Weekend	22,943 (28%)	4002 (30%)	<0.001	26,945 (28%)
Chest pain	19,147 (23%)	2972 (22%)	<0.001	22,119 (23%)
Impaired consciousness, paralysis	13,129 (16%)	1549 (12%)	<0.001	14,678 (15%)
Unclear problem	11,048 (14%)	2565 (19%)	<0.001	13,613 (14%)
Others	4416 (5%)	1045 (8%)	<0.001	5461 (6%)
Respiratory problem	8309 (10%)	1133 (8%)	<0.001	9442 (10%)
Road collision	4049 (5%)	994 (7%)	<0.001	5043 (5%)
Seizure	3990 (5%)	753 (6%)	<0.001	4743 (5%)
Accident, (not traffic related)	3860 (5%)	435 (3%)	<0.001	4295 (5%)
Sick child	1048 (1%)	267 (2%)	<0.001	1315 (1%)
Potential fatal	2838 (3%)	393 (3%)	0.001	3231 (3%)
Bleeding, non-traumatic	1713 (2%)	114 (1%)	<0.001	1827 (2%)
Abdominal pain, back pain	1440 (2%)	93 (1%)	<0.001	1533 (2%)
Psychiatry, suicide	1117 (1%)	169 (1%)	0.31	1286 (1%)
Allergic reaction	1420 (2%)	206 (2%)	0.089	1626 (2%)
Alcohol intoxication, overdose	1458 (2%)	317 (2%)	<0.001	1775 (2%)
Unconscious adult	2805 (3%)	446 (3%)	0.50	3251 (3%)

^a^Median (25%, 75%); *n *(%)

^b^Wilcoxon rank sum test; Pearson’s *χ*^2^ test

### Mortality and admission

Non-conveyed patients had a crude 30-day mortality rate of 2% compared to 6% among conveyed patients, *p* < 0.001. Admission to the hospital within 3 h of the primary contact was observed in 11% (1507 cases) of the non-conveyed patients. In the cohort of the initially conveyed patients, 95% of the patients (77,310 patients) were still admitted after three hours following their initial contact, *p* < 0.001. Admission within 48 h was seen in 3010 cases (22%) of non-conveyed patients compared to 77,853 cases (95%) in conveyed patients, *p* < 0.001 (Table 2).

**Fig. 2:**
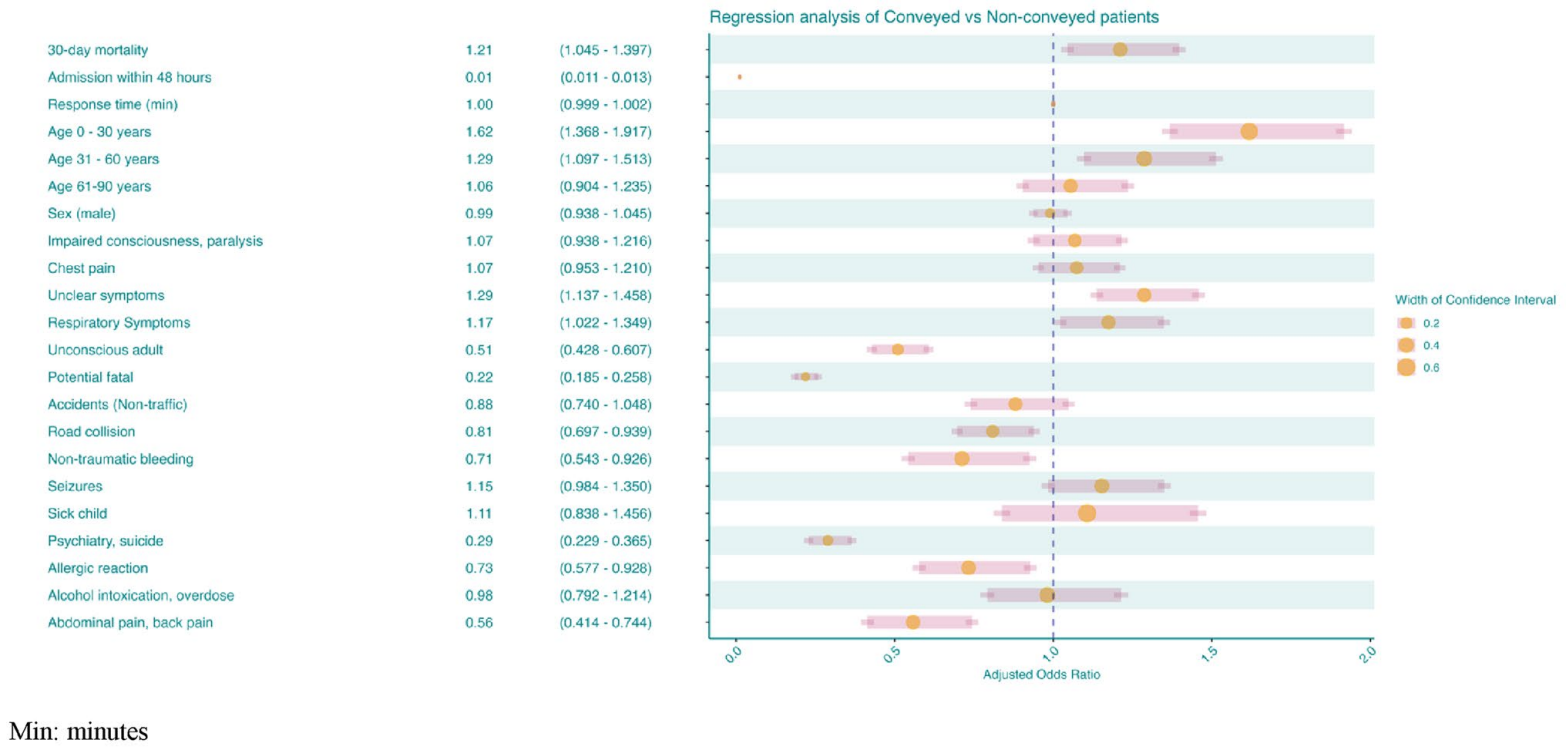
Adjusted multivariable analysis of non-conveyed and conveyed patients (reference)

Within the multivariable analysis, non-conveyed patients showed a decreased likelihood of presenting with potentially fatal symptoms (aOR 0.22 [95% CI 0.19–0.26)] or presenting symptoms related to psychiatry and suicide, aOR 0.29 (95% CI 0.23–0.37). Non-conveyed patients were less likely to be unconscious, aOR 0.51 (95% CI 0.43–0.61). Non-conveyed patients showed an increased likelihood of presenting with unclear symptoms aOR 1.29 (95% CI 1.14–1.46). Additionally, the risk of mortality was increased within the non-conveyed patients, aOR 1.21 (95% CI 1.05–1.40) and finally, non-conveyed patients showed a decreased likelihood of admission within 48 h from the initial call, aOR 0.01 (0.01–0.01) (Fig. 2).

### Non-conveyed patients

**Fig. 3:**
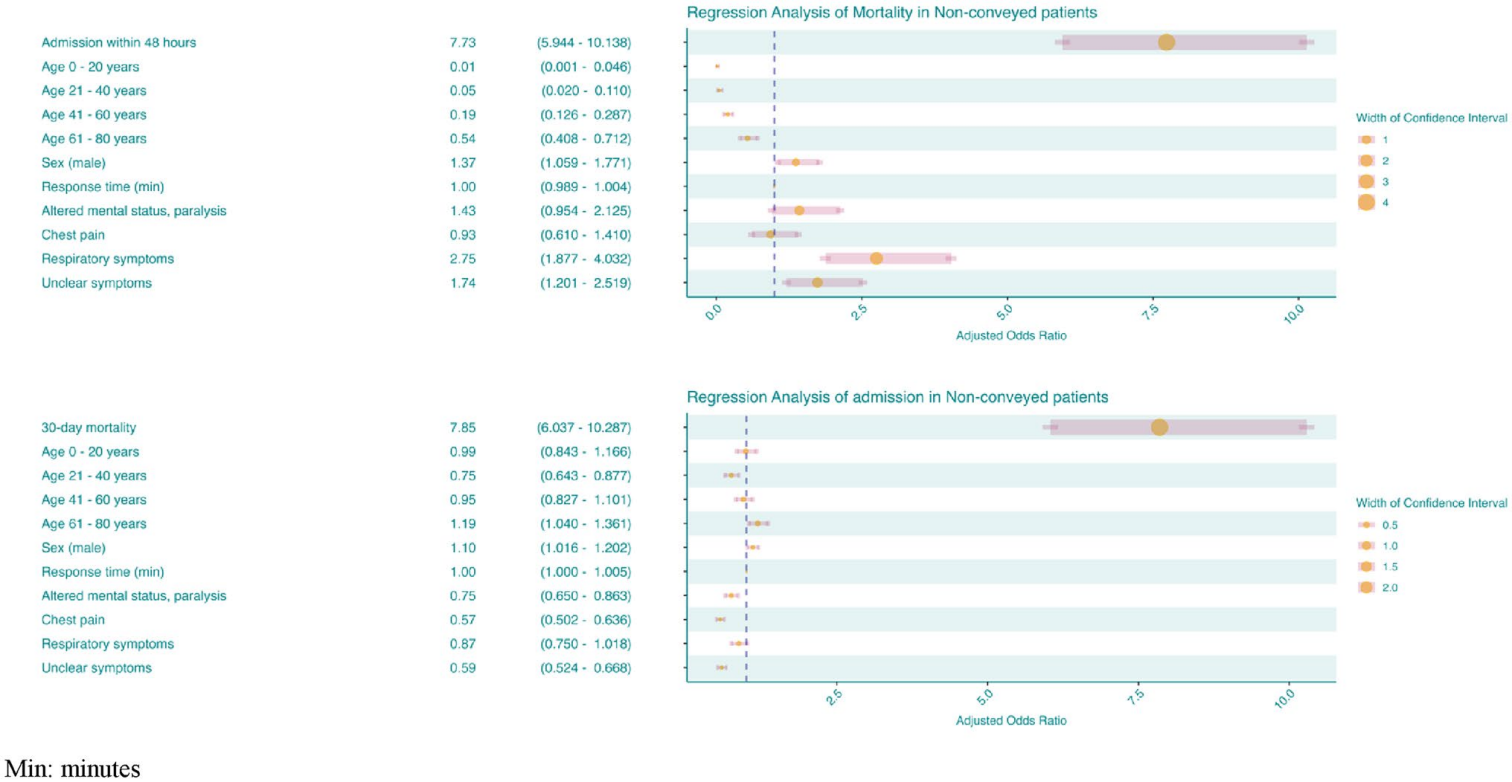
Adjusted multivariable analyses of mortality and admission among non-conveyed patients

Being categorized by the EMDC as having “Respiratory symptoms” and/or “unclear symptoms” was associated with an increased likelihood of mortality, aOR 2.75 (95% CI 1.88–4.03) and aOR 1.74 (95% CI 1.20–2.52). Similarly, male sex was associated with an increased likelihood of mortality aOR 1.37 (95% CI 1.06–1.77). The strongest predictor for mortality was admission within 48 h, aOR 7.73 (95% CI 5.94–10.14) (Fig. 3).

Patients aged 61 to 80 were most likely to be admitted within 48 h, aOR 1.19 (95% CI 1.04–1.36). Additionally, male sex was associated with an increased likelihood of admission within 48 h aOR 1.10 (95% CI 1.01–1.20). On the contrary, patients presenting with “altered mental status” or “paralysis”, “chest pain”, or “unclear symptoms” were less likely to be admitted at any time within 48 h. Respiratory symptoms did not contribute significantly to this model (Fig. 3).

### All admitted patients

**Fig. 4:**
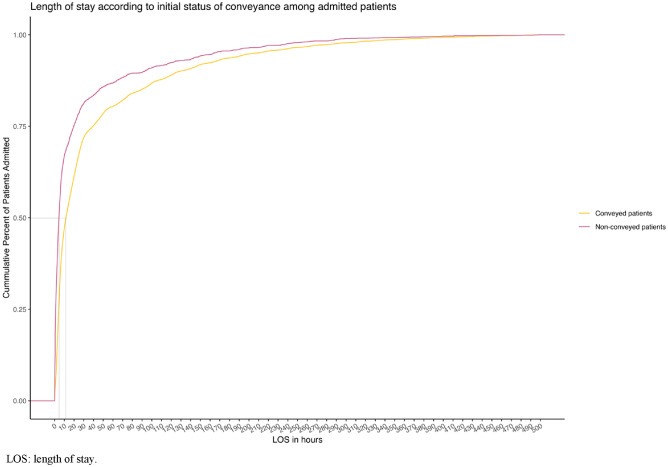
Length of stay in admitted conveyed and non-conveyed patients

In total, 3147 cases (65.4%) of the initially non-conveyed patients were discharged within 6 h from admission compared to 26,979 cases (33.4%) of the conveyed patients, *p* < 0.001. Furthermore, 50% of initially non-conveyed patients were admitted for 4 h or less. In comparison, 50% of conveyed patients were admitted for 11 h or less (Fig. 4). The initially non-conveyed patients, who were later admitted, had a median length of stay (LOS) of 4.4 h (IQR 1.1–20.2) in contrast to the median length of 12.1 h for conveyed patients (IQR 4.2–20.2), *p* = 0.001.

The most frequent chief complaint among admitted patients was “chest pain” which accounted for 19,184 cases (20.1%). “Impaired consciousness” accounted for 13,267 cases (13.9%) and unclear problems for 11,019 (11.6%). With respect to discharge diagnoses, the most frequent were symptoms, signs and abnormal clinical and laboratory findings, not elsewhere classified, followed by injury, poisoning and certain other consequences of external causes accounting for 24,992 cases (30.9%) and 11,970 cases (14.8%). The third most frequent discharge diagnosis was cardiovascular disease, with 11,185 cases (13.8%). One-fourth of patients presenting with chest pain as the chief complaint received a discharge diagnosis of cardiovascular disease, while approximately half of the patients with respiratory problems as their chief complaint received a discharge diagnosis of respiratory disease (Supplementary Fig. 1).

## Discussion

In this retrospective population-based cohort study, the associations between hospital admission and mortality among non-conveyed patients following high-acuity dispatches were assessed. An overall non-conveyance rate of 14% was identified, and the main finding was that less than one in four non-conveyed patients was admitted to a hospital within 48 h following their initial contact with the EMS. The 30-day mortality rate in non-conveyed patients was 2%, including patients who had previously been declared as having a terminal illness.

Different EMS systems have divergent approaches to non-conveyance. Further, variations in regional legislation on non-conveyance and education among EMS personnel and inconsistencies in study designs challenge the comparison of findings. The non-conveyance rate within the present study is comparable to what has previously been reported based on global data on the general population [2]. The fact that this study only included high-acuity dispatches should, of course, be considered when interpreting these results. The Danish EMS is mostly staffed by physicians and paramedics, but in Region Zealand, the personnel in the vehicles are solely paramedics and emergency technicians, which makes the comparison more feasible with other countries’ EMS.

The findings of this study indicate notable distinctions between non-conveyed and conveyed patients across various facets. Non-conveyance generally involved younger patients within this study, which is aligned with reported findings from previous studies [4, 20]. This observation suggests an association between increasing age and the need for medical interventions or examinations at the hospital. Previous studies support the equitable distribution of sex between non-conveyed and conveyed patients [2, 4, 20]. Notably, when considering the non-conveyed population, male sex was associated with a 42% increase in the risk of mortality. Further, male sex was associated with a 17% increased risk of admission. On-scene-time was significantly longer for non-conveyed patients; this finding aligns with previous research and may be explained by the fact that non-conveyance decisions are often multifaceted and time-consuming [21, 22]. Data regarding these associations within non-conveyed patients remain scarce, and focus on confounding is limited; thus, further research is required prior to interpretation in a meaningful context.

From a patient safety standpoint, it holds significance that most non-conveyed patients exhibited an absence of subsequent admissions and mortality, consistent with prior research findings [6, 11, 23]. Among non-conveyed patients, the crude mortality rate was 2%. Despite variations in conditions, dispatch priory level and time intervals of outcomes, the mortality rate observed in this study aligns with comparable studies, wherein reported mortality rates have ranged from 0 to 2.3% [6, 11, 12, 23]. Yet, the adjusted risk of mortality within non-conveyed patients was significantly higher compared to conveyed patients. This could be explained by the unknown proportion of patients declared terminally ill within the non-conveyed cohort [12]. Nonetheless, this finding stresses the need for additional research to ascertain possible confounders and risk factors associated with this correlation. Finally, a high-acuity dispatch would be sent to patients with suspected cardiac arrest, according to the Danish Index [16]. If the patient is legally deceased, there may be a risk of misclassification regarding registering non-conveyance.

In the context of the patient-centred approach, it is imperative to consider both patient satisfaction and patient autonomy. Non-conveyance decision-making may be influenced by patients’ preferences; however, it is noteworthy that the body of research within this domain remains scarce [24].

Data on the LOS within the initially non-conveyed population are limited. This study found that most patients from both cohorts had relatively short lengths of stay, as visualised in Fig. 4. For future research, the divergence regarding the LOS in admitted initially non-conveyed patients is relevant, in line with the economic implications inherent in these findings.

The most frequent chief complaint was “chest pain”, followed by “impaired consciousness” and “unclear problems”, with the latter accounting for 11.6% of all patients. Previous studies have demonstrated that patients presenting with unclear or unspecific complaints are older [25]. Further, it has been shown that a significant proportion of those presenting with unspecific complaints sustained underlying severe conditions associated with increased morbidity and mortality [26]. When considering the discharge diagnoses of this study, the most prevalent diagnosis was symptoms, signs and abnormal clinical and laboratory findings, not elsewhere classified. This study found that approximately 50% of patients with unclear complaints were given an unspecific discharge diagnosis. This is consistent with previous findings by Ibsen et al. [27]. There has been increased focus on stratification of patients presenting with chest pain since studies have shown that a majority of these patients suffer from low-risk conditions [28, 29]. An interesting pattern was revealed concerning patients presenting with chest pain since only 20% resulted in a diagnosis of cardiovascular disease, whereas 60% resulted in unspecific diagnoses (Fig. 3)*.* These findings underscore the challenges within this field and expose the need for further research.

### Limitations

A strength of this study was the large sample size and population-based study design. Further, the comprehensiveness of the employed registries ensured thorough follow-up, mitigating the potential for selection bias. Additionally, equitable availability of prehospital health care services ensures that the process of inclusion remains unaffected by variations in socioeconomic status. The latter may pose implications for the decision of non-conveyance, and future research could benefit from adjusting for this potential confounder. While the data for this study were comprehensive, the fact that the reason for non-conveyance was not available is a limitation. Further, Region Zealand EMS operates with trained paramedics and physician support available for conferences prior to non-conveyance decisions, which may limit generalizability to regions with different EMS competencies available. Caution is advised when extrapolating findings beyond Denmark, highlighting the need for further research. Further, the non-randomized design represents an inherent susceptibility to selection bias, potentially influencing the extent to which the findings can be extrapolated. In this present study, it was not possible to clarify the causes behind non-conveyed decisions; in addition to this, there was no information regarding patients’ perspectives on non-conveyance. Within the group of initially non-conveyed patients, 11% were admitted within 3 h of the non-conveyance decision. Unfortunately, it was beyond the scope of the present study to evaluate this subgroup. However, it could have been relevant to investigate the means of transport in this group. Additionally, this study only investigated high-acuity dispatches, which challenge the generalisability to the general population.

## Conclusion

In conclusion, non-conveyed patients represent a noteworthy proportion of patients assessed by the EMS by a high-acuity dispatch. Less than one in four non-conveyed patients were subsequently admitted within 48 h. Although a low mortality rate was observed in this study, an increased risk of mortality was identified within the non-conveyed population. Future research should focus on evaluating the epidemiology within the non-conveyed population to discern potential confounding variables and inherent risk factors within this group.

## Supplementary Information

Below is the link to the electronic supplementary material.

Supplementary file1 (DOCX 938 KB)

## Data Availability

Data supporting the findings from this study are available from the Danish Patient Safety Authority. Restrictions apply to the availability of these data, which were used under license for the present study, thus, data are not publicly available. Data are, however, available from the authors upon reasonable request and with permission of the Danish Patient Safety Authority.

## Abbreviations

aOR: Adjusted odds ratio
CI: Confidence interval
IQR: Interquartile range
ED: Emergency department
EMS: Emergency Medical Services
EMDC: Emergency Medical Dispatch Centre
LOS: Length of hospital stay
