# Supplementary material for: Treat and release: an observational study of non-conveyed high-acuity dispatches in a Danish emergency medical system
Source: Intern Emerg Med. 2024 May 15;19(8):2283–92. doi: 10.1007/s11739-024-03618-3 (PMC11582337; doi:10.1007/s11739-024-03618-3)
Supplement: Supplementary file 1 — Supplementary file1 (DOCX 938 KB) [file 11739_2024_3618_MOESM1_ESM.docx]

**
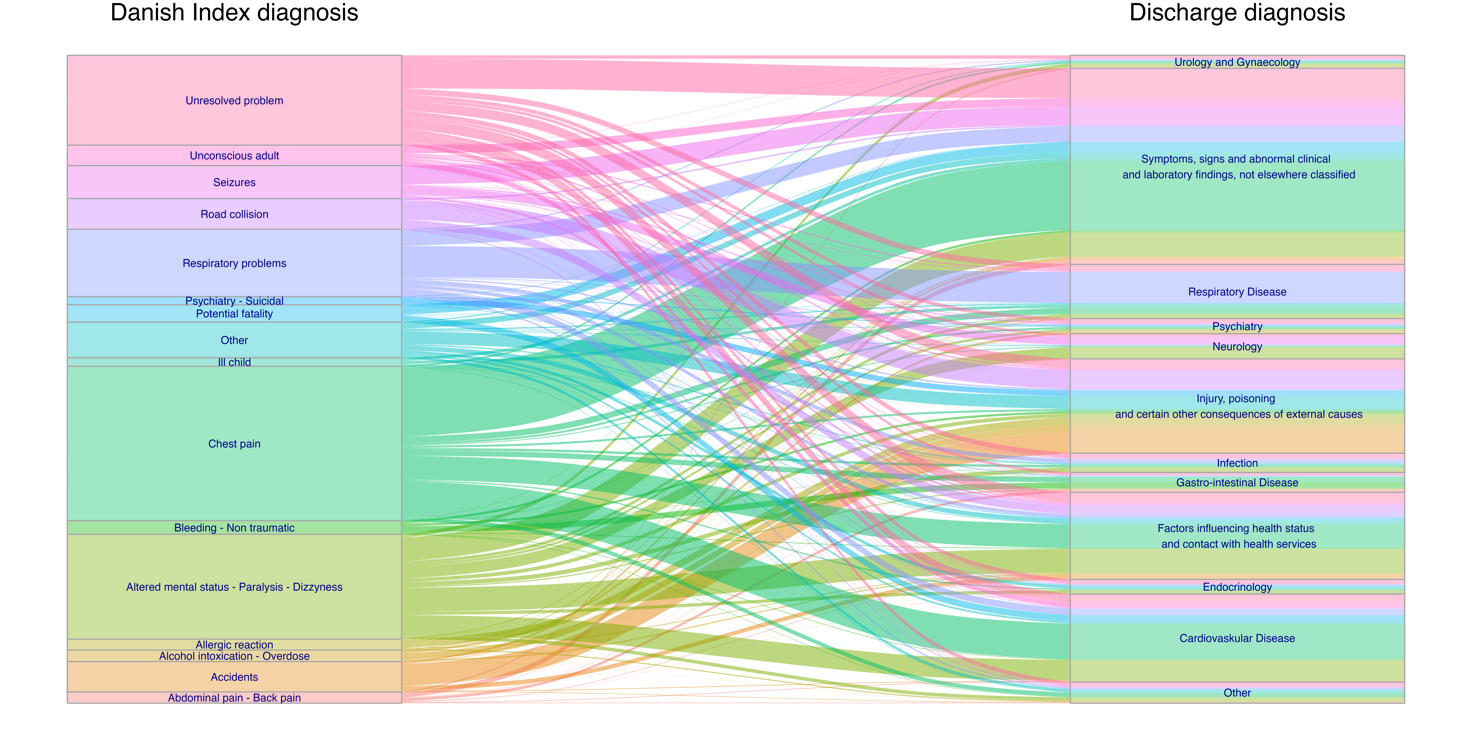
Supplementary file 1: Sankey diagram of chief complaints according to the Danish Index and the corresponding discharge diagnosis among admitted patients.**
